# Neural coding of monaural and binaural intensity at low stimulus frequencies

**DOI:** 10.1186/1471-2202-16-S1-P157

**Published:** 2015-12-18

**Authors:** Zbynek Bures, Petr Marsalek

**Affiliations:** 1 Dept. of Electric Engineering and Computer Science, College of Polytechnics, Jihlava, 586 01, Czech Republic; 2 Inst. of Experimental Medicine, Academy of Sciences of the Czech Republic, Prague, 142 20, Czech Republic; 3 Dept. of Pathological Physiology, First Faculty of Medicine, Charles University in Prague, 128 53, Czech Republic; 4 Czech Technical University in Prague, Zikova 1903/ 4, 166 36, Czech Republic

## 

At low sound frequencies, spikes in the auditory nerve (AN) are phase-locked to the stimulus waveform. For sinusoidal stimuli, spike occurrences are restricted to the positive half-waves of the sound cycles, which constraints possible values of spike rates and their variability. As the spike rates are used for representation of sound intensity, the just noticeable differences (JNDs) of sound level and of inter-aural level difference (ILD) will be influenced. Due to the lack of appropriate AN data, we explore the topic using a computational model [1].

**Figure 1:**
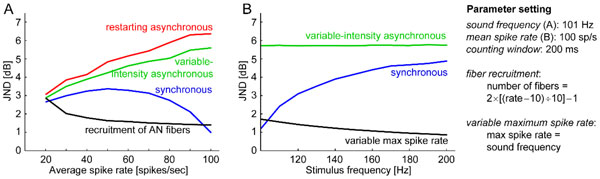
**Just-noticeable differences of sound level depending on spike rate (**A**) and sound frequency (**B**)**.

Three possible types of spike trains are considered: a synchronous, an asynchronous spike train with variable intensity, and a periodically restarting (with sound period) asynchronous process. Generally, the synchronous process leads to several times lower JNDs than the other processes. The JNDs depend on further parameters: longer counting windows or higher number of converging fibers result in smaller JNDs, larger spike timing jitter (random displacement of spikes from their ideal position) slightly increases the JNDs. Notably, the dependence of JNDs on mean spike rate is in contradiction to psychophysical observations that report monotonic decrease of JND of intensity with increasing sound pressure level, the so called near-miss to Weber's law [2]. Even with ideal phase-lock and no jitter, the simulated JND of the synchronous algorithm has a bell shape, the other two algorithms give monotonically increasing function (Figure 1A, colored lines). Recruitment of more AN fibers with rising intensity overcomes this discrepancy; a simulation of this mechanism yields results well matching the psychophysical results (Figure 1A, black line).

Another discrepancy of the modeling results with psychophysics is the simulated JND increasing with frequency, given a constant spike rate (Figure 1B), while psychophysical JNDs decrease with frequency. However, if we assume that maximum spike rate is in some way limited by the frequency (e.g., at most one spike can occur in each sound cycle) and that a given intensity range (e.g., 30 dB) is always mapped to the available range of spike rates (e.g., between zero and sound frequency), then the slope of the rate-intensity function increases and the JND decreases with frequency. The results show that intensity coding has specific properties at low sound frequencies, deserving more detailed electrophysiological studies than what is available in experimental literature today.
